# Pulmonary Evaluation in Children with Post-COVID-19 Condition Respiratory Symptoms: A Prospective Cohort Study

**DOI:** 10.3390/jcm12216891

**Published:** 2023-11-01

**Authors:** Einat Shmueli, Ophir Bar-On, Ben Amir, Meir Mei-Zahav, Patrick Stafler, Hagit Levine, Guy Steuer, Benjamin Rothschild, Lior Tsviban, Nofar Amitai, Miri Dotan, Gabriel Chodick, Dario Prais, Liat Ashkenazi-Hoffnung

**Affiliations:** 1 Pulmonology Institute, Schneider Children’s Medical Center of Israel, 14 Kaplan Street, Petach Tikva 49202, Israel; ophirbo@clalit.org.il (O.B.-O.); meir_zahav@clalit.org.il (M.M.-Z.); patrickst@clalit.org.il (P.S.); hagitlevine@clalit.org.il (H.L.); guysh2@clalit.org.il (G.S.); binyaminro@clalit.org.il (B.R.); liorts@clalit.org.il (L.T.); nofaram2@clalit.org.il (N.A.); mirido@clalit.org.il (M.D.); prais@tauex.tau.ac.il (D.P.); 2 Faculty of Medicine, Tel Aviv University, Tel Aviv 69978, Israel; benami@clalit.org.il (B.A.); hodik_g@mac.org.il (G.C.); liat.ashkenazi@clalit.org.il (L.A.-H.); 3 Department of Day Hospitalization, Schneider Children’s Medical Center of Israel, Petach Tikva 49202, Israel

**Keywords:** post-COVID-19 condition, long COVID, pulmonary function tests, lung clearance index, SARS-CoV-2

## Abstract

**Background:** Studies on post-COVID-19 condition (PCC) in adults have shown deterioration in pulmonary function tests (PFTs), mainly a diffusion limitation. Among the pediatric population, data are scarce. **Aim:** To characterize PFTs in children with PCC, including changes over time. **Methods:** A prospective longitudinal study of children with defined PCC and respiratory complaints who were referred to a designated multidisciplinary clinic from 11/2020 to 12/2022. **Results:** Altogether, 184 children with a mean age of 12.4 years (SD 4.06) were included. A mild obstructive pattern was demonstrated in 19/170 (11%) at presentation, as indicated by spirometry and/or positive exercise challenge test and/or reversibility post bronchodilators, only three had a previous diagnosis of asthma. Lung volumes and diffusion were normal in all but one patient (1/134, 0.7%). Exhaled nitric oxide levels were elevated in 32/144 (22%). A total of 33 children who had repeated PFTs had normal or near-normal PFTs on follow-up testing, including seven (21.2%) who had mild obstructive PFTs at presentation. Multivariate analysis identified older age [OR 1.36 (95% CI:1.07–1.75)], specific imaging findings (prominent bronchovascular markings (OR 43.28 (95% CI: 4.50–416.49)), and hyperinflation (OR 28.42, 95% CI: 2.18–370.84)] as significant predictors of an obstructive pattern on PFTs. **Conclusions:** In children with PCC and respiratory symptoms, the most common impairment was a mild obstructive pattern; most were without a history of asthma. Improvement was witnessed in long-term follow-up. In contrast to the adult population, no diffusion limitation was found. Empirical periodic inhaler therapy may be considered in children with factors associated with PFT abnormalities.

## 1. Introduction

Since coronavirus disease 2019 (COVID-19) has emerged, many studies have reported long-lasting symptoms after recovery, commonly known as ‘Long COVID’ or ‘post COVID-19 condition’) PCC(, the latter being the preferred terminology by the World Health Organization (WHO) [1]. The definition of PCC in children includes a history of confirmed SARS-CoV-2 infection, with at least one persisting physical symptom for a minimum duration of 12 weeks after initial testing that cannot be explained by an alternative diagnosis. The symptoms have an impact on everyday functioning, may continue or develop after COVID infection, and may fluctuate or relapse over time [2]. These symptoms include dyspnea, chronic cough, and chest tightness, as well as cognitive dysfunction and extreme fatigue [3,4].

Studies monitoring post-COVID pulmonary function tests (PFTs) in adults have shown altered respiratory function, with abnormal diffusion capacity being the most prevalent finding, appearing in 40% to more than 50% of patients in most studies [5,6,7]. These studies found restrictive patterns to be much less common and only a small minority of patients demonstrated an obstructive pattern [6].

It is well known that acute COVID-19 infection in children is less severe than in adult patients [8,9,10], with cough as a dominant feature of acute illness [11], but little is known about the prevalence and severity of pediatric PCC [12,13,14].

To date, only a few studies monitoring PFTs in children with PCC and respiratory symptoms have been published [12,13,14,15]. These studies included mostly children with asymptomatic mild acute COVID-19 infection, with a follow-up of up to 8 months post infection, and showed mixed results, ranging from no abnormalities to an obstructive pattern seen in a large proportion of patients. In a systemic review and meta-analysis by Martino et al. the [16] authors have found that children can develop persistent respiratory symptoms after SARS-CoV-2 infection; however, the methodological variabilities of the analyzed studies did not allow them to provide firm conclusions about the rate, type and best diagnostics for children with persistent respiratory symptoms. Furthermore, long-term follow-up data on children are scarce and guidelines regarding the management of these cases are lacking.

In November 2020, a designated multidisciplinary clinic was implanted at our tertiary pediatric center for children with complaints associated with PCC symptoms [17].

In the current study we followed children presenting to this designated clinic with respiratory complaints after recovery from microbiologically confirmed COVID-19 who prospectively performed PFTs. The aims of our study were to evaluate whether PFT abnormalities are present in pediatric PCC, as seen in the adult population, and to characterize those abnormalities. In addition, the study aimed to evaluate longitudinal changes in PFTs over time in this population. Characterization of the respiratory abnormalities in these children may elucidate the underlying pathophysiology and potentially provide a basis for treatment for this chronic condition.

## 2. Methods

### 2.1. Study Design and Population

This prospective cohort study included children referred to a designated multidisciplinary clinic for PCC at Schneider Children’s Medical Center of Israel (SCMCI), a tertiary pediatric center, between November 2020 and December 2022. Inclusion criteria included the following: (a) children ≤18 years of age AND (b) SARS-CoV-2 infection microbiologically confirmed by PCR during acute infection or by subsequent serology AND (c) symptoms suggestive of PCC >12 weeks from acute illness that cannot be explained by an alternative diagnosis AND (d) respiratory complaints (exercise intolerance, dyspnea, cough, etc.) [2].

All referred patients underwent an evaluation including baseline chest X-ray and a structured interview which included demographic data, medical history, and acute COVID-19 infection symptoms, as well as assessment of PCC symptoms and their impact on daily activities. The presence of atopy was determined by anamnesis as well as review of electronic medical records and included personal or familial history of atopic manifestations (dermatitis, asthma, or allergic rhinitis). Past respiratory comorbidities were evaluated based on self-report and medical records.

**Figure 1:**
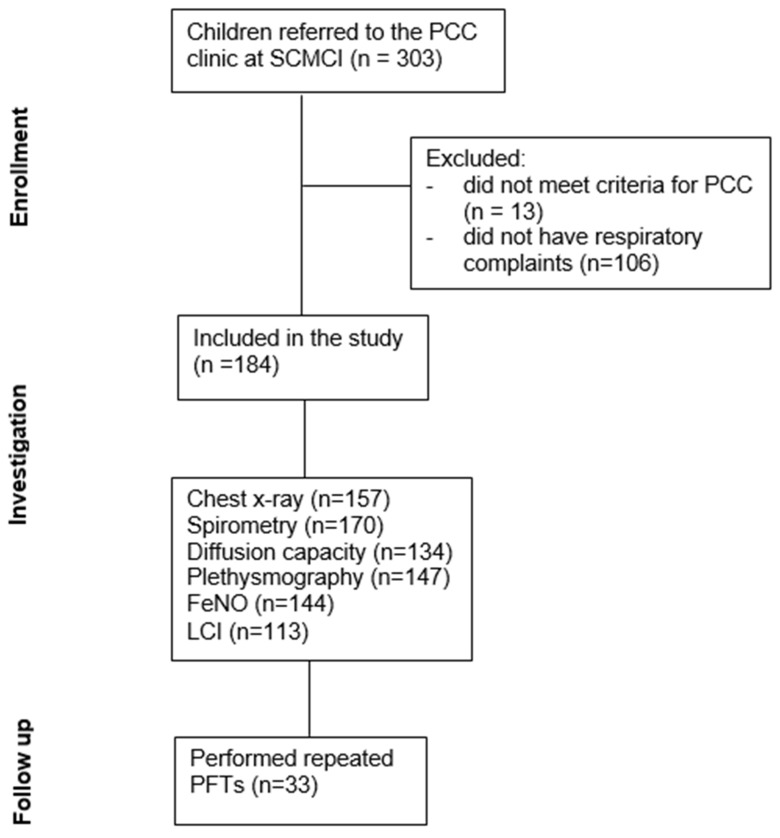
Consort flow diagram of the study.

The subset of children with cardio-respiratory complaints (e.g., dyspnea, cough, chest tightness, and exercise intolerance) underwent additional investigation, including electrocardiography (ECG) echocardiography, and comprehensive PFTs according to age, which included baseline spirometry, exercise challenge test (ECT), bronchodilator response, lung plethysmography, diffusion capacity, fractional exhaled nitric oxide (FeNO), and multiple breath washout (MBW). In cases of severe continuous complaints and based on clinical judgment, a cardiopulmonary exercise test (CPET) was performed. Following this investigation, children with abnormal PFTs were assessed by a pediatric pulmonologist; if indicated, specific treatment was prescribed and further investigation, including repeated PFTs and methacholine challenge testing (MCT), was performed when clinically appropriate (Figure 1).

### 2.2. Microbiology

A positive SARS-CoV-2 PCR test was defined according to the Israeli Ministry of Health guidelines [18]. Testing methods included the Real-Time Fluorescent PCR kit (BGI) and the SARS-CoV-2 PCR kit (Seegene, Seoul, Republic of Korea). Anti-spike receptor-binding domain IgG antibodies were measured using an in-house enzyme-linked immunosorbent assay (The Central Virology Laboratory of the Ministry of Health at Sheba Medical Center, Tel Hashomer, Israel) until mid-March 2021 and the SARS-CoV-2 IgG II Quant assay (Abbott) thereafter.

### 2.3. Pulmonary Function Testing

PFTs were measured in the lung function laboratory of the SCMCI’s pulmonary institute.

#### 2.3.1. Spirometry and Exercise Challenge

Spirometry was obtained using Smart PFT UI (Medical Equipment Europe GmbH, Hammelburg, Germany) according to the American Thoracic Society (ATS)/European Respiratory Society (ERS) consensus guidelines [19].

ECT was performed while running on a treadmill for 6 min, with speed and slope adjusted to achieve a target heart rate of (220 − age) × 0.8 for a minimum of 4 min, according to ATS/ERS guidelines [20,21]. Heart rate and oxygen saturation were monitored continuously during running. Post-exercise spirometry was performed at 1, 3, 5, 10, 15, and 20 min after ceasing exercise. Any fall in FEV_1_ of 10% or greater from baseline FEV_1_ was considered as positive [20]. A bronchodilator (4 puffs of salbutamol metered-dose inhaler 100 mcg) was administrated when the ECT was positive or when FEV_1_ was <95% predicted. Reversibility was defined by a rise of 10% or greater in FEV_1_ [21].

#### 2.3.2. Plethysmography and Diffusion Capacity

Plethysmography and diffusion capacity for carbon monoxide were obtained using Smart PFT body box (Medical Equipment Europe GmbH, Hammelburg, Germany) according to the ATS/ERS guidelines [22]. Carbon monoxide diffusion was corrected for alveolar volume and hemoglobin level, indicated as KCO. Spirometry and lung volumes were expressed as percent of predicted normal values with the use of the equations of Polgar and Varuni [23]. KCO percent predicted was calculated as described by Weng and Levinson [24].

#### 2.3.3. Exhaled Nitric Oxide Levels (FeNo)

FeNo was measured using a chemiluminescence analyzer (CLD 77 AM; EcoMedics AG, Duernten, Switzerland) according to ATS/ERS recommendations [25]. Normal values were considered ≤20 parts per billion (ppb) for single-breath or ≤8 ppb for multiple-breath test.

#### 2.3.4. Lung Clearance Index (LCI)

MBW, expressed as the Lung Clearance Index (LCI), was performed with an ultrasonic flow meter (Spiroson 1; EcoMedics AG, Switzerland) according to ATS/ERS consensus statement [26]. Normal values were considered < 7.91 [27].

#### 2.3.5. Methacholine Testing

MCT was obtained using Smart PFT UI (Medical Equipment Europe GmbH, Germany) using a triple-dose accelerated protocol of fresh methacholine solutions in normal saline solution [28]. A positive methacholine test was defined as provocative concentration causing 20% decline in FEV_1_ (PC20) of <4 mg/mL [21]. A bronchodilator (4 puffs of salbutamol metered-dose inhaler 100 mcg) was administrated when the methacholine challenge test was positive or when FEV_1_ at the end of the test was <80% predicted.

### 2.4. Statistical Methods

Descriptive statistics were used to define the demographic and clinical characteristics of the cohort. Multivariate logistic regression was used to assess the association between clinical patient characteristics (pre-defined independent variables selected on the basis of clinical evaluation) and an obstructive pattern on pulmonary function testing (dependent variable). The Wald test was used for CI calculation. A probability value of ≤0.05 was considered significant. Results are presented based on the full data set. Interactions were systematically searched for. Data were analysed using IBM SPSS Statistics for Windows, version 27.0 (IBM Corp. Released 2020 and R Core Team, 2016, Armonk, NY, USA).

## 3. Results

### 3.1. Patient Characteristics

Altogether 303 Children were assessed for PCC at our multidisciplinary clinic between November 2020 and December 2022. Of those, 184 reported respiratory symptoms at enrolment and thus were included in this study.

#### 3.1.1. Demographic and Clinical Parameters

The mean age of the participants was 12.6 years (SD 4.06, range 2–18 years), with 33/184 children (17.9%) under 10 years old; of those, 111 (60.3%) were female and mean BMI% was 60.8% (SD 31.7%). Most children had no underlying diseases (137, 74.5%). Previous asthma diagnosis was reported in 11 (5.4%) and depression/anxiety disorders in eight (4.3%). In total, 17 (9.2%) were diagnosed with attention deficit disorder (ADHD), and 24 (13%) were competitive athletes. Most participants had mild or asymptomatic acute SARS-CoV-2 infection (170, 92.4%), with only five children (2.7%) hospitalized due to O_2_ saturation <94%, with disease defined as “severe” by the National Institutes of Health (NIH) [29] (Table 1).

#### 3.1.2. Post-COVID Respiratory Symptoms and Consequences

Evaluation was done at a mean time of 25.1 weeks (IQR 13.7–34.9) after acute COVID infection. The most common respiratory symptom was dyspnea (136, 73.9%), followed by chest pain (89, 48.4%), and cough (19, 10.3%). In all, 104 (56.5%) reported mild functional impairment, with moderate and severe impairment in 74 (40.2%) and six (3.3%), respectively. Activities of daily living were significantly affected in 116 (63%) and 142 (77.2%) reported impaired physical activity (Table 2).

### 3.2. Cardio-Pulmonary Evaluation

- Among patients who performed PFTs, 170/184 (92%) performed spirometry, with a mean FEV_1_ of 92.6% (SD 10.6%, Table 3); 19/170 (11%) patients had FEV_1_ < 80%, of which FEV_1_/FVC was <80% in three of them, one with a rheumatologic disease and two previously healthy, and one with a personal history of atopy; the latter two also had a positive ECT and all had significant reversibility post bronchodilators.
- ECT was performed by 133 children (Table 3), of whom four had a positive test; all four had complete post bronchodilator reversibility, three were previously healthy, and one had a background gastrointestinal disease; none had a history of asthma, but three had personal atopy. Eleven other children had negative ECT but a rise of ≥10% in FEV_1_ post bronchodilators; one had asthma and six of the remaining 10 had either a personal or familial history of asthma. All the participants who performed ECT maintained normal O_2_ saturations during the test.
- Following baseline spirometry with FEV_1_ < 90% or FEV_1_/FVC < 90%, 11 children were evaluated for bronchodilator responsiveness, of whom three showed FEV_1_ improvement of ≥10%. All three had no previous history of asthma or atopy.
- Overall, 19/175 (11%) children had an obstructive pattern, as indicated by FEV_1_/FVC <80%, and/or positive ECT, and/or post bronchodilator reversibility—of whom only one had a previous asthma diagnosis.
- Altogether, 144 children underwent FeNO testing (Table 3), of whom 32 (22%) had elevated values, and 4/34 had a previous diagnosis of asthma as well as personal atopy. Another four children with elevated FeNO levels also demonstrated an obstructive pattern on spirometry: two with FEV1/FVC < 80% and two more with positive ECT. All four had personal atopy, and 14 others had either a personal or family history of atopy.
- In all of the 147 children who performed plethysmography lung volume testing, TLC was normal (≥80% predicted, Table 3); RV/TLC > 150% was found in 27 (18%), of whom one had a history of asthma.
- In all, 111 children performed MBW test, with a mean LCI of 7.48 (SD 0.9, Table 3); 27/111 (24.3%) had a value of >7.9, of whom two had known asthma, seven more had personal atopy and three had familial atopy; none had a history of prematurity and baseline spirometry was normal in all but one, who also had a significant post bronchodilator reversibility.
- Of the 134 children who performed a lung diffusion test (Table 3) KCO was >75% predicted in all but one patient with a rare genetic syndrome, post kidney transplantation, without a previous known lung disease.
- Overall, abnormal PFTs—including an obstructive pattern, elevated FeNO, air trapping, elevated LCI, or diffusion limitation—were documented in 85 children (46.2%); of those, 20/85 had at least two abnormal tests, and 17/20 demonstrated an obstructive pattern.

**Table 3:** Pulmonary function tests in 184 children with post-COVID-19 condition and respiratory complaints.

Test	*n*	Result, Mean (SD)
FEV_1_% predicted	170	92.6% (10.6)
FVC% predicted	170	95.9% (11.4)
FEV_1_/FVC%	170	90.38% (6.9)
FEV_1_% post exercise	133	91.7% (10.6)
FEV_1_% post BD *	96	93.9 (9.5)
FeNO		
SBT **, ppb (normal < 8 ppb)	128	15.1 (14.6)
MBT **, ppb (normal < 20 ppb)	16	13.5 (8.4)
TLC% predicted	147	105.2 (12.9)
RV/TLC%	146	118.5 (32.7)
KCO% predicted	134	104.3 (14.2)
LCI	113	7.48 (0.9)
MCT ** (*n* = 7)		
Positive/borderline	3	
Negative	4	

Either post ECT or post spirometry alone. * BD = Bronchodilator. ** SBT = Single Breath Test, MBT = Multiple Breath Test, MCT = Methacholine Challenge Test, CXR = Chest X-ray.

- As part of their assessment, chest X-ray was performed on 157/184, of whom results were normal in 122 (78%), while 13 (8%) exhibited peribronchial cuffing, seven (4%) prominent bronchovascular markings, and 10 had other abnormalities, as detailed in Table 4.

**Table 4:** Chest X-ray results in 157 children with post-COVID-19 condition and respiratory complaints.

Result	*n* (%)
Normal	122 (77.7%)
Peribronchial cuffing	13 (8.3%)
Prominent bronchovascular markings	7 (4.5%)
Residual pulmonary infiltrate	6 (3.8%)
Hyperinflation	2 (1.3%)
Enlarged cardiac silhouette	1 (0.6%)
Minimal effusion unilateral	1 (0.6%)

- ECG testing was done in 167 children; nine (5.4%) had abnormal results that were previously identified and were not COVID-related.
- Echocardiography was performed in 145 children, showing mild abnormalities in six (4.1%), all detected previous to SARS-CoV-2 infection.
- CPET testing was performed in six children due to continuous severe complaints of exercise intolerance at a median time of 38.8 weeks after acute COVID infection (range 32.8–115.8 weeks); five were previously healthy individuals and one had known asthma. In all cases, CPET demonstrated normal cardio-respiratory function.

#### 3.2.1. Pulmonary Evaluation in Children with Moderate to Severe Acute COVID-19

- Among the 9 children with moderate acute COVID-19 infection according to the NIH criteria [29], one demonstrated an obstructive pattern on PFTs without a previous asthma diagnosis; three had elevated FeNO values, of whom two had either a personal or a familial history of atopy; two had elevated LCI values, one with known asthma.
- Among the 5 children with severe acute COVID-19 infection, none had abnormal spirometry, two had elevated FeNO values, of whom one had a personal history of atopy, and two had elevated LCI values. One child, post kidney transplantation, had diffusion limitation.

#### 3.2.2. Pulmonary Evaluation in Children Previously Vaccinated against SARS-CoV-2

Overall, three children (1.6%) in our cohort were previously vaccinated against COVID-19. All had normal spirometry upon evaluation, one had mildly elevated FeNO and one had an elevated LCI.

#### 3.2.3. Multivariate Logistic Regression Analysis for Factors Associated with an Obstructive Pattern on Pulmonary Function Testing

Upon evaluation of predicting factors for an obstructive pattern, defined as FEV_1_/FVC < 80% and/or positive ECT and/or reversibility post bronchodilators, the following were found to have a significant predictive value: older age (OR 1.36 (95% CI:1.07–1.75)), CXR findings of prominent bronchovascular markings (OR 43.28 (95% CI: 4.50–416.49) and hyperinflation (OR 28.42, 95% CI: 2.18–370.84), as detailed in Table 5.

Multivariate logistic regression was used to assess the association between clinical patient characteristics and an obstructive pattern on pulmonary function testing. Results are presented based on the full data set. There are missing data for 7 patients. Interactions were systematically searched for.

### 3.3. Interventions and Drug Therapy

In all, 17 children with an obstructive pattern on PFTs were evaluated by a pediatric pulmonologist and 16 of them received a trial of inhaler therapy with a combination of inhaled corticosteroid (ICS) and long-acting beta agonists (LABA).

### 3.4. Pulmonary Long-Term Follow-Up

A total of 33 children performed repeated PFTs at least 30 days after the first test, due to previous abnormal PFTs and/or continuous respiratory complaints. The second evaluation was done at a mean time of 13.6 weeks (IQR 6.3–21.7 weeks) after the first evaluation. At baseline evaluation 7/33 (21.2%) had abnormal PFTs, demonstrating a mild obstructive pattern. Seventeen of the 33 (51.5%) were followed in the pulmonary institute, of which 16 received inhaler therapy as previously detailed. Upon follow-up, all 33 children had normal or near-normal PFTs on repeated testing. Four of them underwent CPET with normal results. All 33 had reported clinical improvement in their respiratory symptoms upon follow-up.

## 4. Discussion

This prospective cohort study of children with PCC respiratory symptoms provides comprehensive data on their PFTs and the change over time. Respiratory function abnormalities were seen in 85 (46.2%) of the participants, of whom 17 (20%) had reversible obstructive pattern—most of them without known asthma. Repeated PFTs 1–6 months later have demonstrated normalization of PFTs in all children re-tested after presenting with an obstructive pattern, most of them (16/17) treated with inhaled corticosteroids. Furthermore, unlike adults with PCC [5,6,7], diffusion capacity and exercise O_2_ saturation remained within normal limits in our study population.

Previous reports on PCC in the pediatric population [9,10,15] suggest a different pathogenesis than in adult PCC: while in adults, pulmonary interstitial or vascular changes are the cause of diffusion limitation [5], it seems that in children the disease does not cause these changes. One possible mechanism of respiratory PCC in children, which may explain the majority of symptomatic children with normal PFTs, is autonomic dysfunction—as suggested by Morrow et al. [30]. This mechanism is also supported by the study by Knoke et al. [14] in which the PFTs of children with PCC and respiratory complaints, including MBW, body plethysmography, and diffusion capacity testing, were all within normal limits.

Another mechanism, which may explain the reversible obstructive pattern seen in some children in our cohort as well as in the studies by Leftin Dobkin et al. [13] and by Palacios et al. [12], might be post-inflammatory changes causing airway hyper-responsiveness. Post-viral wheezing is a known phenomenon after RSV and Rhinovirus infection in early childhood [31] and similar mechanisms might also explain airway hyper-responsiveness in our study and support the notion of inhaled beta-agonists and corticosteroids as possible treatment for PCC respiratory symptoms. A meta-analysis by Tau et al. has demonstrated a large proportion of co-infection with *influenza* virus among children with COVID-19 compared to adults [32]; this fact might also contribute to the high prevalence of the presumed post-inflammatory reaction causing airway hyper-responsiveness.

As opposed to the studies by Leftin Dobkin et al. [13] and Palacios et al. [12], which reported high rates of children with asthma diagnosis suffering from PCC respiratory symptoms, only 2.5% of the children in our cohort had a previous diagnosis of asthma. These results are in keeping with some reports on PCC in adults, which found a low asthma rate in their cohorts [33], but there are also reports suggesting a higher incidence of PCC among asthmatics [34]. One theory is that enhanced Th- 2 immune responsiveness, while having a protective effect against acute-COVID, increases the risk of PCC [35]. This theory might be supported by the fact that 45% of our cohort had a history of personal or familial atopy and also by the high FeNO values seen in 22% of the children performing the test, most, but not all, with an atopic background (22/32, 69%).

A high proportion of competitive athletes was found in our study, in keeping with the study by Palacios et al. [12]. This may be explained by the fact that physical activity is a known trigger of bronchial hyperreactivity, and while most children kept a sedentary lifestyle during the COVID-19 outbreak, competitive athletes were still engaged in physical activity during the quarantines or early after social distancing provisions were withdrawn [36].

Repeated PFTs in children with continuous respiratory complaints and normal baseline PFTs did not change in the follow-up test. Furthermore, in all children who presented with reversible obstructive pattern at first visit, repeated PFTs had demonstrated normalization, including negative MCT. Palacios et al. also reported clinical improvement as well as improvement in spirometry and 6 min walk test in their patients at follow-up, although to a smaller degree [12]; other observational studies also showed clinical improvement over time [37].

As older age and specific abnormalities on chest X-ray were significant predictors of obstructive PFTs in our cohort, it might be beneficial to perform PFTs, mainly ECT, in this subset of patients, as well as to consider inhaler therapy for these children.

Treatment options for children with PCC remain extremely limited. In the current cohort, 16/17 children with obstructive pattern on PFTs received inhaler therapy with ICS-LABA; all improved over time. Clinical improvement was also noted in the 17 children who did not receive inhaler therapy. Although treatment was not specifically addressed in the present study, it may be suggested that bronchodilators or inhaled corticosteroids can be a transitory treatment option for the period of severe respiratory symptoms.

The main limitation of our study is the lack of prior PFTs in study participants, so some may have had undiagnosed asthma prior to COVID-19; however, these children had no respiratory symptoms prior to COVID (13% were participating in competitive sports) and also, in those with repeated testing, improvement in their PFTs was apparent with time. Another limitation is that follow-up testing was only available for some of the cohort; nevertheless, in the majority PFTs improved, supporting the notion of a temporary phenomenon.

In conclusion, PCC respiratory symptoms were associated with mild abnormal findings in almost half of the children tested; of them, 20% had an obstructive pattern that resolved with time. Therefore, bronchodilators or inhaled corticosteroids can be considered as a transitory treatment option for the period of severe respiratory symptoms. Unlike adults, PFTs did not show diffusion limitation. Future studies should focus on the effectiveness of various treatments for this population.

## Figures and Tables

**Table 1:** Demographic and clinical characteristics of 184 children with post-COVID-19 condition and respiratory symptoms.

Age, mean ± SD, years	12.64 ± 4.06
Female, *n* (%)	111 (60.3%)
BMI%, mean ± SD	60.8% ± 31.7%
Underlying disease, *n* (%) *	45 (24.5%)
Asthma	11 (5.4%)
Depression/anxiety	8 (4.3%)
Gastrointestinal disease	5 (2.7%)
Rheumatologic disease	4 (2.2%)
Immunodeficiency **	4 (2.2%)
Neurological impairment ***	4 (2.2%)
Nephrological disease	3 (1.6%)
Other ****	9 (4.9%)
Prematurity (<37 weeks)	4 (2.2%)
Atopic background by history, *n* (%)	
Personal	50 (27.2%)
Familial	32 (17.4%)
Attention deficit disorder (ADHD), *n* (%)	17 (9.2%)
Competitive athletes, *n* (%)	24 (13%)
Severity of acute COVID-19 illness according to NIH *****, *n* (%)	
Asymptomatic	9 (4.9%)
Mild	161 (87.5%)
Moderate	9 (4.9%)
Severe	5 (2.7%)

* Including 3 children with two underlying diseases. ** Neurological impairment, including developmental delay and epilepsy. *** Immunodeficiency, including congenital immunodeficiency, solid organ transplantation, and hematopoietic stem cell transplantation. **** Other, including migraine/headache, kidney disease, and hematologic disease. ***** National Institutes of Health (NIH) [29].

**Table 2:** Chronic respiratory symptoms in 184 children with post-COVID-19 condition.

Respiratory Complaint, *n* (%)	
Dyspnea	136 (73.9%)
Chest pain	89 (48.4%)
Cough	19 (10.3%)
Functional status, *n* (%)	
Mild	104 (56.5%)
Moderate	74 (40.2%)
Severe	6 (3.3%)
Influence ADL *, *n* (%)	116 (63%)
Physical activity effected, *n* (%)	142 (77.2%)

* ADL = Activities of Daily Living.

**Table 5:** Multivariate analysis of predicting factors for obstructive pattern on pulmonary function testing.

	OR	95%CI	*p*-Value
Age	1.36	1.07–1.75	0.014
Chest pain	2.97	0.88–10.08	0.080
CXR			
Prominent bronchovascular markings	43.28	4.50–416.49	0.001
Hyperinflation	28.42	2.18–370.84	0.011
Peribronchial cuffing	4.06	0.84–19.77	0.082

## Data Availability

The data presented in this study are available on request from the corresponding author. The data are not publicly available due to privacy restrictions.
